# Supplementary material for: Autonomic Dysfunction during Acute SARS-CoV-2 Infection: A Systematic Review
Source: J Clin Med. 2022 Jul 4;11(13):3883. doi: 10.3390/jcm11133883 (PMC9267913; doi:10.3390/jcm11133883)
Supplement: Supplementary file 1 [file jcm-11-03883-s001.zip › jcm-1786084-supplementary.pdf]

Supplementary materials

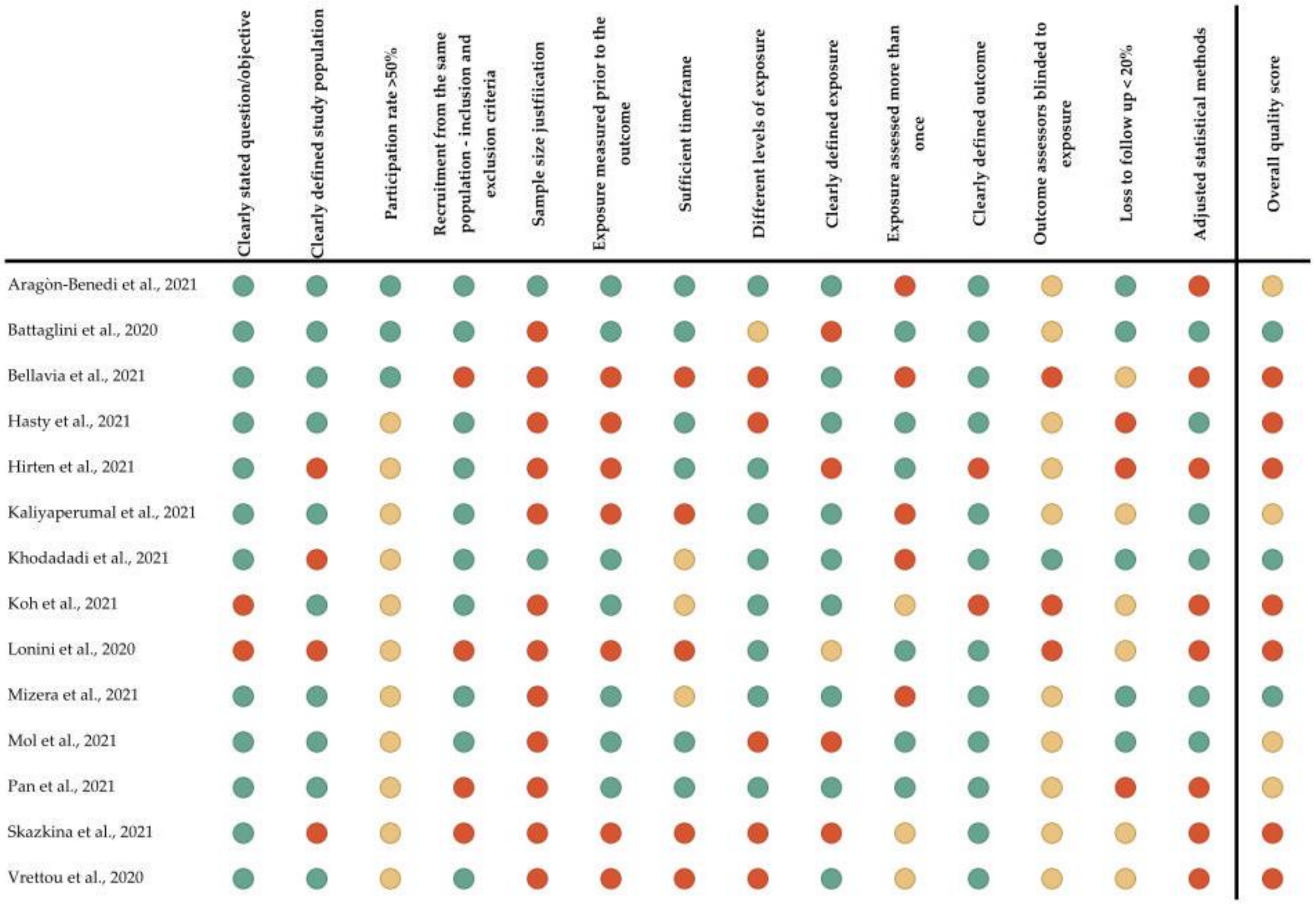

Figure S1. Quality assessment of cohort and cross-sectional studies according to the "Study Quality Assessment Tool" issued by the National Heart, Lung, and Blood Institute. Color legend: Green, yes; Yellow, not applicable, not reported, or cannot determine; red, no.

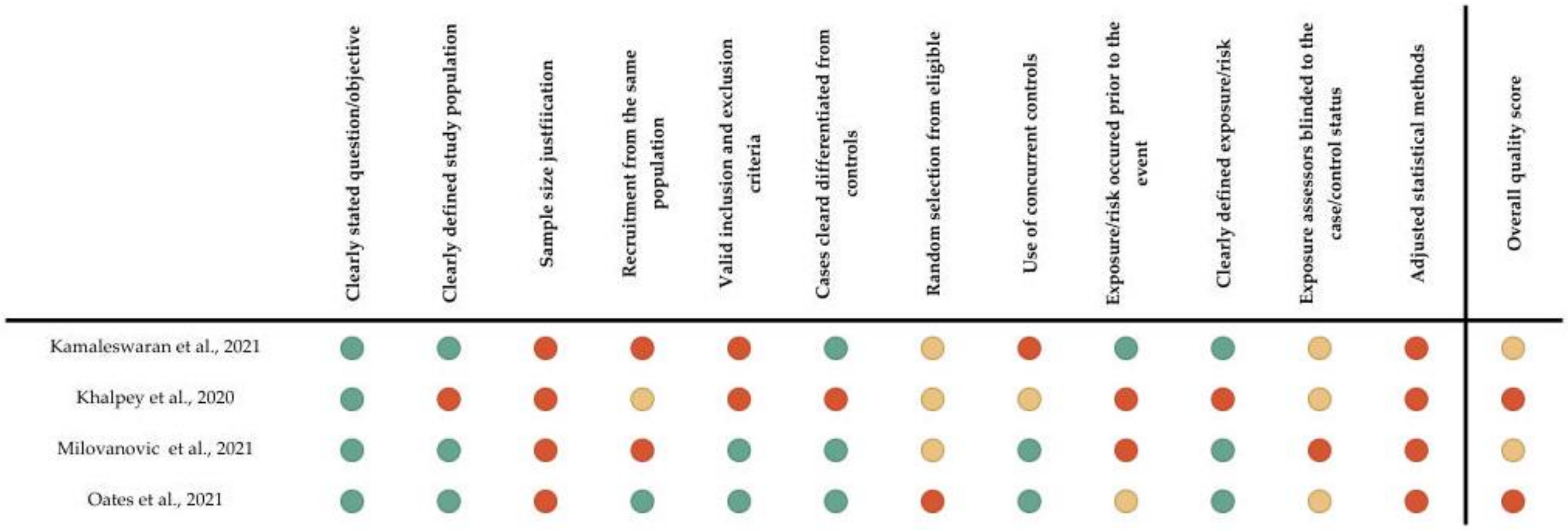

Figure S2. Quality assessment of case-control studies according to the "Study Quality Assessment Tool" issued by the National Heart, Lung, and Blood Institute. Color legend: Green, yes; Yellow, not applicable, not reported, or cannot determine; red, no.

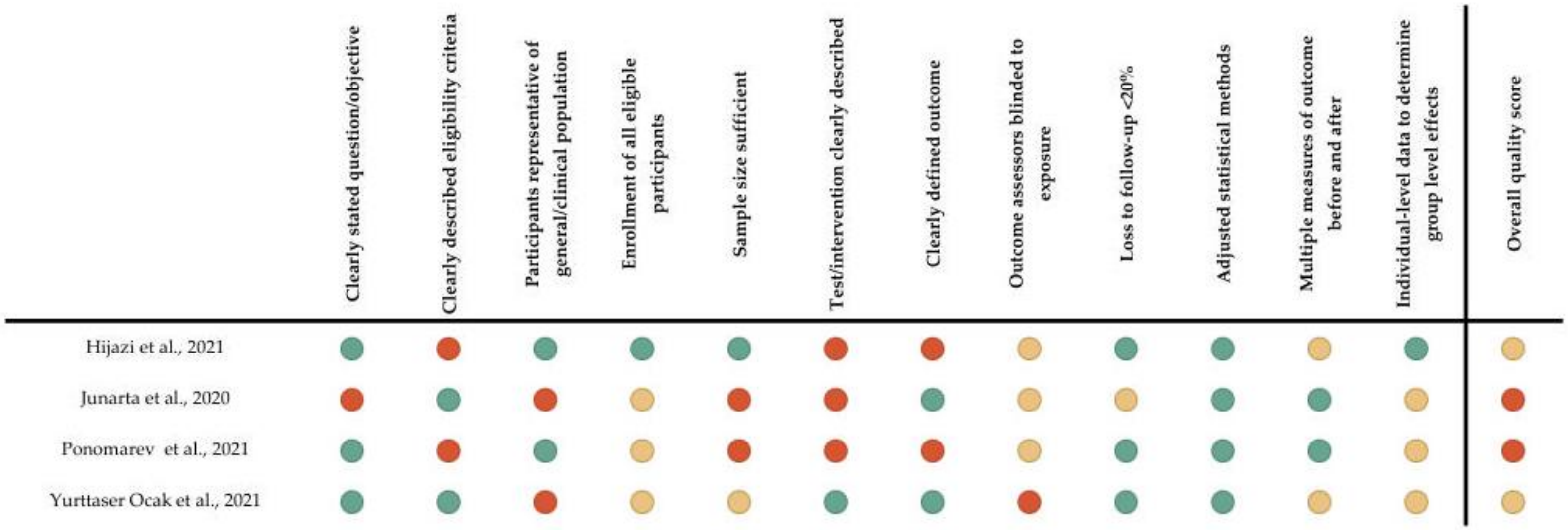

Figure S3. Quality assessment of pre-post studies according to the "Study Quality Assessment Tool" issued by the National Heart, Lung, and Blood Institute. Color legend: Green, yes; Yellow, not applicable, not reported, or cannot determine; red, no.
